# Rho GTPase Signaling in Health and Disease: A Complex Signaling Network

**DOI:** 10.3390/cells10020401

**Published:** 2021-02-16

**Authors:** Cord Brakebusch

**Affiliations:** Biotech Research and Innovation Center (BRIC), University of Copenhagen, Ole Maaløes Vej 5, 2200 Copenhagen, Denmark; cord.brakebusch@bric.ku.dk

Rho GTPases are a family of small G-proteins of the Ras superfamily. The ubiquitously expressed Rho GTPases RhoA, Rac1, and Cdc42 are major regulators of the actin cytoskeleton, with essential functions in cell migration. However, Rho GTPases also regulate other processes than actin polymerization and are involved in the regulation of proliferation, differentiation, polarity, and stemness. These effects are mediated by a large number of effector molecules, which are often co-regulated by other pathways, resulting in a complex signaling network and considerable cell-type specificity. Not only with respect to effectors but also to the regulatory side, Rho GTPase signaling is complex due to the large number of activating (guanine nucleotide exchange factors (GEFs)) and inactivating (GTPase activating proteins (GAPs) and guanine nucleotide dissociation inhibitors (GDIs)) molecules, which in turn are controlled by many different signaling pathways. This complexity explains why many aspects of Rho GTPase signaling are poorly understood, in particular with respect to the in vivo function. 

In this Special Issue entitled Rho GTPase Signaling in Health and Disease, we collected review articles and original papers that shed light on specific aspects of Rho GTPase function during development and disease, often with a focus on certain Rho GTPases. 

The role of Rho GTPase signaling in cancer has been investigated thoroughly for many years. Whereas Rho GTPases initially were simply considered invasion-promoting, pro-oncogenic molecules, recent research including cancer genome sequencing has resulted in a more differentiated view of Rho GTPase functions in cancer, revealing both promoting and inhibiting effects and high cancer-type specificity. In this Special Issue, De et al. review the role of Rac1 in solid cancers [1], whereas Melzer et al. focus on Rac1B [2], an alternatively spliced isoform of Rac1 with a different functional and regulatory profile. Zhang et al. propose the Rho GTPase Cdc42 as a target for breast cancer therapy and provide evidence for the involvement of long non-coding RNAs in the regulation of Cdc42 during tumor formation [3]. Voena and Chiarle describe how both gain- and loss-of-function mutations of Rho GTPases could contribute to the development of lymphoma [4].

In addition to cancer, Rho GTPase signaling has emerged as a key regulatory pathway in a number of other diseases. Guo et al. describe the role of Rho GTPase signaling in autism spectrum disorders, where 20 Rho GTPase regulator or effector genes are suggested to be disease-associated [5]. Mulherkar and Tolias discuss how targeting RhoA/ROCK signaling could help in therapy for traumatic brain injuries [6], and de Curtis examines the non-redundant neuronal developmental and disease-related functions of Rac3 which is a close relative to Rac1, but tissue-specifically expressed [7]. In glucose metabolism and metabolic disorders, aberrant Rho GTPase function appears to play a major role, as Møller and Sylow present in their review [8]. The role of Rho GTPases in vascular disease is described by Strassheim et al. [9]. Although these articles cannot cover all disease-related functions of Rho GTPase, they illustrate the widespread importance of Rho GTPase signaling in human disease.

Several articles in this Special Issue describe tissue-specific roles of Rho GTPase signaling. Bros et al. analyze RhoA as a crucial regulator of innate and adaptive immunity, where it regulates the migration and activation of immune cells [10]. Rho GTPase function in the peripheral nervous system is described by Kalpachidou et al., describing neuronal development as well as neuroregeneration [11]. The spatiotemporal regulation of Rho GTPases enduring the migration of neurons is the topic of a review by Xu et al. [12]. Highly polarized sensory neurons as present, for example, in the cochlea are dependent on Rho GTPase function, as explained in the review article by Ueyama [13]. Rho GTPases are crucial for the formation of blood vessels during development (vasculogenesis), the sprouting of novel blood vessels from pre-existing vessels (angiogenesis) as in wound healing and cancer, and for the maintenance of the mature vessel barrier. Barlow and Cleaver review the scientific literature corresponding to this area in this Special Issue [14]. In this context, the original article by Atat et al. is important, showing the function of RhoG downstream of Rac1 and Cdc42 in the tube formation of vascular endothelial cells [15]. 

Another set of reviews highlights specific effectors and regulators of Rho GTPases. The Cdc42-binding protein Cdc42SE1 is identified as a negative regulator of Cdc42-dependent growth signaling with potential importance in skin cancer by Kalailingam et al. [16]. Bustelo and Rodrigues-Fdez introduce the Vav protein family, which are both GEFs for Rho GTPases and signaling adaptors downstream of protein kinases [17]. Margioatta and Bucci review the cooperated action of Rac1 and Rab proteins with consequences for diseases [18]. Finally, the function of p190RhoGAP and its two isoforms, p190A and p190B, are described by Heraud et al. [19].

More than three decades after their recognition as master regulators of the actin cytoskeleton, Rho GTPases and their signaling remain an exciting and active research area with many important open questions and implications in many different diseases.
